# The Epidemiology of the Long-Term Care Needs and Unmet Needs of Older Veterans in the United States

**DOI:** 10.3390/jcm14124219

**Published:** 2025-06-13

**Authors:** Sandra Garcia-Davis, WayWay M. Hlaing, Denise C. Vidot, Daniel J. Feaster, Jared Hansen, Ben J. Brintz, Orna Intrator, Luci K. Leykum, Erin D. Bouldin, Ranak B. Trivedi, Polly H. Noel, Stuti Dang

**Affiliations:** 1 Miami VA Healthcare System, Geriatric Research, Education & Clinical Center GRECC, Miami, FL 33125, USA; 2 Department of Public Health Sciences, University of Miami, Miami, FL 33136, USA; whlaing@med.miami.edu (W.M.H.); dvidot@med.miami.edu (D.C.V.); dfeaster@med.miami.edu (D.J.F.); 3 School of Nursing and Health Studies, University of Miami, Coral Gables, FL 33146, USA; 4 Department of Veterans Affairs, Elizabeth Dole Center of Excellence for Veteran and Caregiver Research, San Antonio, TX 78229, USA; jared.hansen@va.gov (J.H.); luci.leykum@va.gov (L.K.L.); erin.bouldin@hsc.utah.edu (E.D.B.); ranak.trivedi@va.gov (R.B.T.); polly.noel@va.gov (P.H.N.); 5 Informatics, Decision-Enhancement and Analytic Sciences Center (IDEAS 2.0), VA Salt Lake City Health Care System, Salt Lake City, UT 84148, USA; ben.brintz@hsc.utah.edu; 6 Division of Epidemiology, University of Utah Health, Salt Lake City, UT 84112, USA; 7 Geriatrics and Extended Care Data Analysis Center (GECDAC), J.J. Peters VA Medical Center, Bronx, NY 10468, USA; orna.intrator@va.gov; 8 Department of Geriatrics & Palliative Medicine, Icahn School of Medicine at Mount Sinai, New York, NY 10029, USA; 9 South Texas Veterans Health Care System, San Antonio, TX 78229, USA; 10 Dell Medical School, University of Texas at Austin, Austin, TX 78750, USA; 11 Department of Internal Medicine, Spencer Fox Eccles School of Medicine, University of Utah, Salt Lake City, UT 84112, USA; 12 Center for Innovation to Implementation (Ci2i), VA Palo Alto Health Care System, Palo Alto, CA 94304, USA; 13 Division of Public Mental Health and Population Sciences, Department of Psychiatry and Behavioral Sciences, Stanford University, Stanford, CA 94305, USA; 14 Division of Geriatrics and Gerontology, Department of Medicine, University of Miami School of Medicine, Miami, FL 33136, USA

**Keywords:** unmet needs, long-term care, Veterans, LTSS, home and community-based services

## Abstract

**Background/Objectives:** Veterans differ in sociodemographic composition and experience higher frequencies of disability than non-Veterans of the same age. Yet the epidemiology of the long-term care needs of Veterans, specifically activities of daily living (ADLs) and instrumental activities of daily living (IADLs), remains an important gap in the literature. The objectives of this study were to (1) characterize Veterans across levels of hierarchy of ADL and IADL support needs; (2) compare Veterans across the degree of need for help, from those who can still “self-manage” to those with an “unmet need”; and (3) identify the types and prevalence of ADL and IADL need combination patterns. **Methods:** This study used cross-sectional data from the 2021 administration of the HERO CARE survey. We included Veterans ages 65+ in our analyses (N = 7424). We calculated the overall weighted descriptive statistics across a hierarchy of ADL and IADL problems and the degree of need for help. One-way ANOVA for continuous variables and Rao–Scott chi-square tests for categorical variables were conducted to examine associations between groups, followed by post hoc pairwise comparisons, as appropriate. **Results:** Veteran respondents mean age was 82.3 (SD: 8.2 years), and most were male, non-Hispanic White, and married. In weighted analyses, more Veterans with both ADL and IADL problems compared to only ADL problems reported food insecurity, missed appointments, low health literacy, and depression. Among Veterans with ADL or IADL problems, 32.3% reported an unmet need for help. Almost a quarter of Veterans with ADL problems reported difficulties performing all eight ADLs (23.9%), and over a quarter of Veterans with IADL problems reported difficulties performing all seven IADLs (31.3%). **Conclusions:** Our findings show that Veterans are demographically and clinically different based on their hierarchy of impairment and degree of need for help. Identifying the patterns and prevalence of ADL and IADL needs among Veterans provides valuable information to align the Veterans Affairs (VA) programs and services with Veterans’ needs.

## 1. Introduction

The Veterans Health Administration (VHA) administers most of the medical care for the United States Department of Veterans Affairs (VA), accounting for more than one-third of all VA expenditures in 2019 [1]. It is the largest integrated healthcare system in the United States and a predominately geriatric system. Approximately half of the VHA patient population is 65 years and older [2]. Older adults’ ability to function independently significantly impacts their quality of life. Therefore, the VA is focused on substantial investments in the planning and development of long-term services and supports (LTSS) to meet the needs of Veterans and enable them to age in place, with a good quality of life [3,4]. LTSS refer to care that is provided in the home, in a community-based setting, or in facilities to assist older adults and people with disabilities with a wide range of services related healthcare and daily activities [4]. LTSS often work in tandem with family caregivers who play a critical role in supporting older adults by providing essential assistance with daily activities, emotional support, and care coordination, often filling gaps left by formal services. Their involvement is key to helping older adults age in place safely and with dignity, delaying or preventing the need for institutional care.

Veterans are over 20 years older on average than non-Veterans [2], meaning that the VHA will experience the impact of aging care recipients years in advance of other healthcare systems. Veterans at a high risk for long-term institutional care, typically characterized by frailty, complex comorbidities, and higher disability levels, potentially face a greater prevalence of long-term care (LTC) needs and unmet needs, as well as differences in the types and patterns of needs than their same age counterparts.

LTC needs are typically defined as difficulty performing activities of daily living (ADLs) (e.g., eating, bathing, toileting, and dressing) and instrumental activities of daily living (IADLs) (e.g., banking, shopping, cooking, and taking medications) [5,6]. LTC is a term that describes the dependence on others for help with daily activities. Older adults also typically have needs across other domains including health information [7], receipt of healthcare [8,9,10,11], and social needs (e.g., food insecurity and transportation) [12,13]. Unmet needs, as they relate to LTC, can be defined as occurring when no assistance is provided to meet older adults’ daily needs, or there is incongruency in the type or quantity of help needed and received. When these needs go unmet, adverse outcomes like hospitalization [12,14,15,16,17], long-term institutional care [18], and death [19] present a higher risk. The unmet needs of older adults are associated with the degree of functional impairment [20], living alone [21], dementia [22], multimorbidity [20], sex [20], and social/caregiving networks [12,23].

While LTC needs among the general older adult population of the United States are widely studied, measures of unmet need are less often utilized [24,25,26,27]. To our knowledge, limited studies to date have evaluated the LTC unmet needs of older community-dwelling Veterans enrolled in the VHA. The few studies on unmet need among Veterans focus on mental health needs and the use of mental health services [28] or focus on Veterans with specific conditions [29]. In the United States, some nationally representative surveys [20,27,30] have sought to understand the unmet needs among older adults, but these surveys are not specific to Veterans enrolled in the VHA or with a focus on those at risk of long-term institutional care. Given that Veterans differ in sociodemographic composition and are more likely to self-report physical disability and experience higher frequencies of comorbid conditions than non-Veterans of the same age [31,32], the epidemiology of the LTC needs and unmet needs of older Veterans is an important gap in the literature to explore [2]. This study focuses on LTC needs specific to ADLs and IADLs because limitations in these areas are strong indicators of long-term institutional care risk, eligibility for VA services such as home and community-based services, and the need for targeted support to help older adults age in place, should that be their preference.

The objectives of this study were to (1) characterize Veterans across levels of hierarchy of ADL and IADL problems, (2) compare Veterans across the degree of need for help, and (3) identify the types and prevalence of ADL and IADL combination patterns.

## 2. Materials and Methods

### 2.1. Study Design and Sample

**Figure A1:**
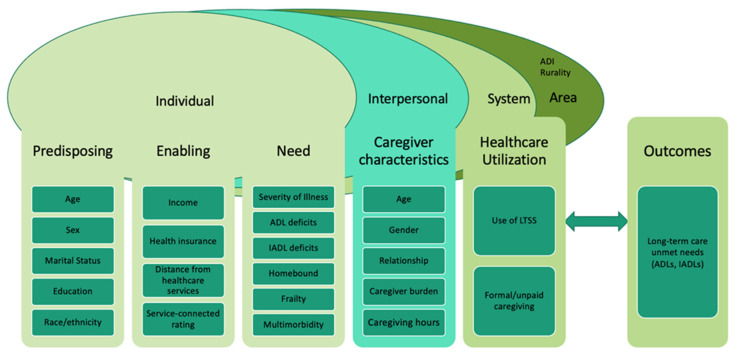
Integrative Andersen’s Behavioral Model of Health Services Use and the Social–Ecological Model.

This study employed an integrative Andersen Behavioral Model of Health Services Use [33] and the Social–Ecological Model [34] to describe Veterans and their needs (Appendix A, Figure A1). Drawing on these models, we considered the multifaceted interchange between the individual-, interpersonal-, community/environment-, and societal-level determinants of health that affect older adults, in general, and Veterans uniquely; for example, the effects of their military service are life-long and multidimensional and are affecting and affected by every level of the Social–Ecological Model from their individual disability to their social relationships, as well as the policy regarding their care. Furthermore, Andersen’s Behavioral Model guides our framing of how Veterans’ predisposing characteristics, enabling resources and health needs, influence their functional limitations and access to LTSS. Current and future policies concerning the availability of LTSS at the societal level trickle down and affect LTC unmet needs at the individual level. With both frameworks in mind, Veterans and caregivers were placed within networked ecosystems, with the understanding that these levels overlap to influence one another. The dynamic nature of the Social–Ecological Model allows for factors to be moved between categories depending on their influence in Andersen’s Behavior Model.

We used cross-sectional data from the 2021 round of the HERO CARE survey, a prospective, multicenter survey of Veterans and their caregivers aimed at understanding the unmet needs of Veterans across multiple health and social domains to inform LTSS [35]. Veterans were eligible to receive the HERO CARE survey if they were community-dwelling and receiving care at one of the four VHA sites (Miami, San Antonio, Salt Lake City, and Palo Alto) or Veteran Integrated Service Network 8 (VISN 8), as determined by having a 2021 Geriatrics and Extended Care Data Analysis Center Core File [35]. Veterans were not eligible if they resided in a nursing home or a community living center, were in hospice, or if they had an undeliverable mailing address. About 704,177 Veterans were eligible, of which we randomly surveyed 20,000 Veterans and oversampled Veterans with a higher predicted long-term institutionalization (PLI) score and a predicted probability of two-year risk of long-term institutional care [36]. The PLI risk score was generated by the VA Geriatrics Extended Care Data Analysis Center using predictive data analytics with VA and Centers for Medicaid and Medicare Services (CMS) data [36]. A detailed description of the HERO CARE protocol is published elsewhere [35].

The HERO CARE Veteran survey included 79 questions on Veteran-specific sociodemographic characteristics not available in VHA records and the following health-related domains: physical health (mobility, daily activities, and frailty), mental health (depression, anxiety, resilience, and substance use), social support (caregiver support and social network), social determinants of health (housing, food and medication insecurity, and transportation), and technology (access, willingness, and ability) [35]. Survey data was combined with the Veterans’ Geriatrics Extended Care Data Analysis Center Core Files for the 2021 fiscal year using the Veterans’ patient internal control number. The overall response rate for Veterans was 40.3% (N = 8056) between July 2021 and January 2022. Our final sample size resulted in 7424 Veterans after excluding those under the age of 65 (n = 410) and those who did not answer any of the ADL and IADL questions (n = 222).

### 2.2. Measures

#### 2.2.1. Item Specific to ADL and IADL Problems

**Figure A2:**
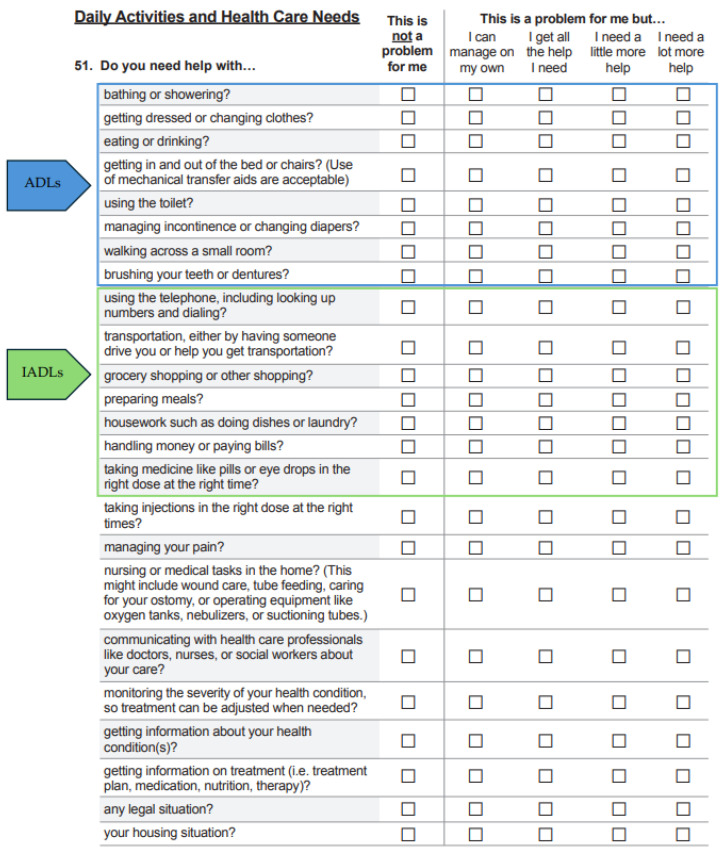
HERO CARE survey questions for ADL and IADL items.

Veterans were asked if they had difficulty with eight ADLs (bathing, dressing, eating, getting in or out of bed, toileting, managing incontinence, walking across a small room, and brushing teeth) and seven IADLs (using the telephone, transportation, grocery shopping, preparing meals, housework, handling money, and taking medicine; Appendix A, Figure A2). For example, Veterans were asked, “Do you need help with bathing or showering?” And the response options were “This is not a problem for me” or “This is a problem for me… (followed by the degree of help needed).” We coded each ADL and IADL to indicate whether or not each one was a problem for the Veteran. Then, we added up the number of problems reported to find the total number of ADL problems (ranging from 0 to 8) and the total number of IADL problems (ranging from 0 to 7).

#### 2.2.2. Hierarchy of ADL and IADL Problems

**Figure 1:**
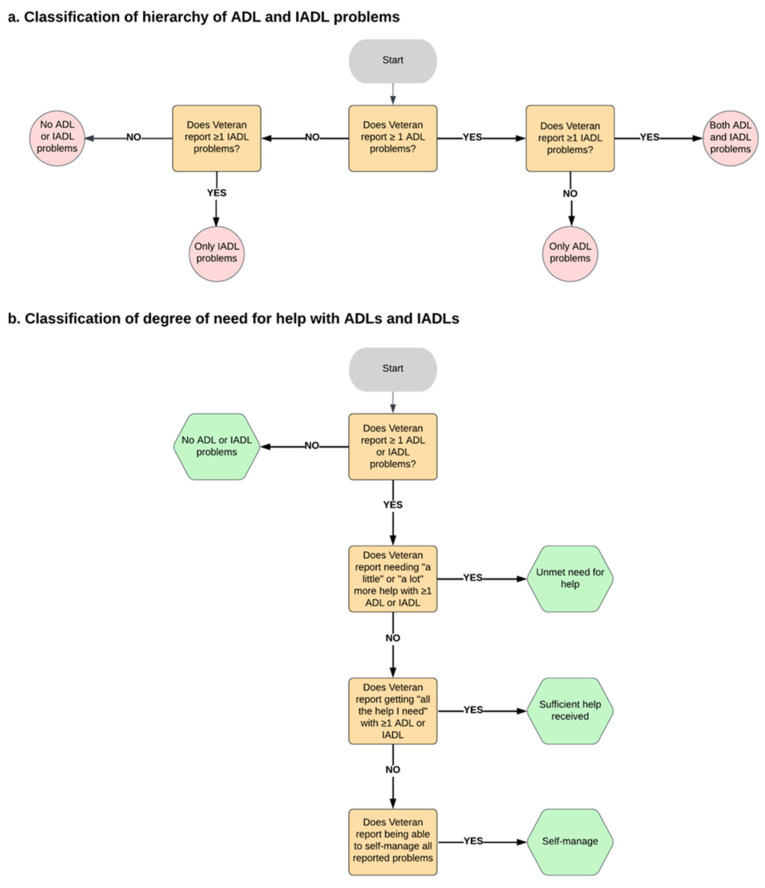
Classification of the hierarchy of ADL and IADL problems and the degree of need for help.

Based on previous studies, IADLs and ADLs have a hierarchical structure where the severity of functional impairment increases from no ADL or IADL problems (preserved function), only IADL problems, and only ADL problems to both ADL and IADL problems [37]. We created a variable for a 4-level hierarchy of ADL and IADL problems using the ADL and IADL problem count variables mentioned previously (Figure 1a) to reflect increasing levels of disability and care dependency. Although this approach has been used in studies of older adults, to our knowledge, it has not been applied to the Veteran population; we adapted it for this study to support the meaningful stratification of Veterans’ LTC needs and unmet needs. Veterans who reported zero IADL problems and zero ADL problems were classified as having no problems. Veterans who reported ≥1 IADL problems but zero ADL problems were classified as IADL only. Veterans who reported ≥1 ADL problems but zero IADL problems were classified as ADL only. Veterans who reported ≥1 ADL problems and ≥1 IADL problems were classified as having both ADL and IADL problems.

#### 2.2.3. Degree of Need for Help with ADLs and IADLs

Veterans who reported having a problem with ADLs or IADLs were instructed to select the degree of help needed—“I can manage on my own,” “I get all the help I need,” “I need a little more help,” or “I need a lot more help”. These options reflect the increasing degree of need for help, with the latter two signifying an unmet need. Identifying the degree of help needed is an important measure because not all older adults require the same level of help. While some may require complete assistance from another person to carry out an activity, others may have problems performing an activity but are still be able to self-manage the activity with, for example, the aid of an assistive device [38].

To place Veterans in the mutually exclusive degree of need for help classes, we employed a set of rules that prioritized the highest degree of need for help reported (Figure 1b): self-manage (the Veteran reports difficulty but manages without help), sufficient help (receives help and reports that it meets their needs), and unmet need (receives no help or insufficient help despite needing it). Self-reported unmet needs offer insights into the perceived adequacy of support and have been shown in prior research to correlate with objective indicators of unmet needs, such as increased hospitalization [12,14,15,16,17]. Lastly, we coded each ADL and IADL item to indicate whether the Veteran reported an unmet need for help. Then, we added up the number of unmet need items to find the total number of ADLs (ranging from 0 to 8) and IADLs (ranging from 0 to 7) for which more help was needed.

#### 2.2.4. Sociodemographic and Clinical Variables

We obtained Veterans’ age, sex, race and ethnicity [39], marital status, rurality (classified using the rural–urban commuting area system, with rural defined as areas with a low population density and limited commuting access to urban centers) [40], area deprivation index (ADI, a measure of neighborhood-level socioeconomic disadvantage) [41], hierarchical condition categories (HCCs) [42], CMS-HCC risk score (an indicator of clinical risk and expected healthcare costs), service-connected rating (extent of service-related impairment) [43], and dementia and substance use disorder diagnosis from GECDAC Core File data [44]. Service-connected disability rating ranged from 0 to 100% for Veterans with a service-connected disability [43]. We also categorized service-connected disability ratings as not service connected, ≤40%, 50–90%, and 100% [45]. HCC indicator variables for dementia (HCCs 51 and 52) and substance use disorders (HCCs 54, 55, and 56) were used [44,46]. The CMS-HCC risk-adjusted score is the sum of the score or weight attributed to each of the demographic factors and HCCs used in the scoring model [42]. The CMS-HCC model is normalized to 1.0, meaning that Veterans would be considered relatively healthy, and therefore less costly, with a risk score less than 1.0 [42].

Self-reported measures from the HERO CARE Veteran survey included sex, marital status, education level, missed appointments due to transportation problems [47], food insecurity [48], medication insecurity [49], health literacy [50], the presence of a caregiver, homebound status [51], possible depression (PHQ-2) [52], and anxiety (GAD-2) [53]. Missing data on self-reported sex and marital status was supplemented by VA EHR, reducing the missing data for sex from n = 178 to n = 4 and for marital status from n = 296 to n = 8.

### 2.3. Statistical Analysis

For each site and risk of institutional care, we calculated the inverse probability of sampling design weights [35]. The weight was calculated for each stratum by dividing the number of patients available to sample by the number sampled. Non-response weights were calculated by estimating the propensity to respond using a gradient-boosted statistical model that accounted for age, race, sex, marital status, independence at home qualification, High-Need High-Risk status, percent service connected, and rurality [35]. We used those propensity scores to calculate average treatment effect weights. A final weight incorporating both the design and non-response weight was used to produce weighted estimates representative of the total population of the sites surveyed. We assessed model diagnostics by examining the distribution of the weight and checking the covariate balance before and after non-response weighting using absolute standardized mean differences visualized with love plots. We calculated weighted descriptive statistics for the full sample, as well as separately by levels of ADL and IADL problems and the degree of need for help. Means and standard errors were reported for continuous variables and frequencies and percentages for categorical variables. We conducted one-way ANOVAs for continuous variables and Rao–Scott chi-square tests of independence for categorical variables. A two-sided *p*-value of <0.05 was considered statistically significant. Significant one-way ANOVA results were followed up by post hoc pairwise comparisons. For post hoc pairwise testing, we applied a Bonferroni correction. All proportions and means used in the analyses were weighted.

To identify the prevalence of different combinations of ADL and IADL problems, respectively, we ranked the most prevalent combinations according to their frequency and weighted percent. We selected the most prevalent combinations using a criterion of ≥2% prevalence in the study population [54]. We used R Statistical Software 4.4.1 (Vienna, Austria: R Foundation for Statistical Computing) and the R twang package to estimate the inverse probability weights and conducted weighted analyses using SAS Survey Proc procedures in SAS Enterprise Guide 8.3 (Cary, NC, USA: SAS Institute Inc.) to account for the complex survey design.

## 3. Results

Table 1 presents the results based on an unweighted sample size of N = 7424 and a weighted population estimate of N = 362,639. The Veterans’ weighted mean age was 76.5 (SE: 0.22). The majority were male (95.8%), non-Hispanic White (69.5%), and married (64.4%); had at least some college education (69.3%); resided in urban areas (80.3%); and were not homebound (91.4%).

### 3.1. Hierarchy of ADL and IADL Problems

Table 1 describes the prevalence of the hierarchy of problems with ADLs and IADLs among older Veterans and compares sociodemographic, clinical, VA and CMS measures, and dependency characteristics across groups. We found that 47.5% of Veterans aged 65 and over had no ADL or IADL problems, 13.2% had only IADL problems, 5.6% had only ADL problems, and 33.7% had both ADL and IADL problems.

Veterans with no ADL or IADL problems were significantly different from those with both ADL and IADL problems in characteristics including age, race and ethnicity, education level, and ADI. Compared to Veterans with both ADL and IADL problems, Veterans with no ADL or IADL problems reported a lower prevalence of medication insecurity, food insecurity, missed appointments due to transportation, low health literacy, substance use disorder, dementia, possible depression, and possible anxiety.

Veterans with only ADL problems, only IADL problems, and both ADL and IADL problems were similar across demographic characteristics, including age, sex, race and ethnicity, marital status, and education level, and most sociodemographic characteristics like rurality, ADI, and medication insecurity.

However, 23.8% more Veterans with both ADL and IADL problems compared to only ADL problems reported food insecurity (29.8% vs. 6.0%, Table 1). We saw similar results for missed appointments due to transportation (17.2% vs. 2.5%), low health literacy (54.7% vs. 20.8%), dementia diagnosis (9.0% vs. 1.2%), and possible depression (31.1% vs. 12.6%). As for dependency characteristics, 33.8% more Veterans with both ADL and IADL problems compared to only ADL problems had a caregiver (65.3% vs. 31.5%), and 17.7% more Veterans were homebound (19.9% vs. 2.2%). Moreover, Veterans with both ADL and IADL problems compared to only IADL problems had a higher prevalence of food insecurity (29.8% vs. 18.8%), dementia (9.0% vs. 3.8%), having a caregiver (65.3% vs. 37.1%), and being homebound (19.9% vs. 6.6%).

### 3.2. Degree of Need for Help

Table 2 describes the prevalence of the degree of need for help with ADLs and IADLs among Veterans and compares sociodemographic, clinical, VA and CMS measures, and dependency characteristics across groups. Over half (N = 5045, 52.5%) of the Veterans reported either ADL or IADL problems or both. Among Veterans reporting ADL or IADL problems, 32.3% reported an unmet need for help with at least one ADL or IADL, 31.6% reported having sufficient help, and 36.1% reported difficulty performing but still being able to self-manage their reported ADL or IADL problems.

The mean number of ADL problems increased from 2.71 among Veterans who could still self-manage to 3.37 among Veterans who had sufficient help and was highest at 4.47 among Veterans with an unmet need for help. The mean number of IADL problems increased from 2.61 among Veterans who could still self-manage to 4.14 among Veterans who had sufficient help and was highest at 5.10 among Veterans with an unmet need for help.

Veterans who could self-manage differed from those who received sufficient help in age (75.0 vs. 78.9 ), sex (female: 10.5% vs. 1.7%), marital status (married: 61.5% vs. 76.5%), low health literacy (29.0% vs. 55.3%), dementia (1.0% vs. 7.2%), and the presence of a caregiver (26.9% vs. 68.3%).

Veterans who could self-manage differed from those who reported an unmet need for help in age (75.0 vs. 78.4), sex (Female: 10.5% vs. 2.8%), food insecurity (19.4% vs. 36.1%), low health literacy (29.0% vs. 63.4%), missed appointments due to transportation (7.0% vs. 28.3%), dementia (1.0% vs. 13.1%), possible depression (18.3% vs. 38.1%), anxiety (15.1% vs. 29.2%), the presence of a caregiver (26.9% vs. 72.1%), being homebound (6.9% vs. 23.7%), and 100% service-connected rating (7.3% vs. 16.0%).

Veterans who received sufficient help differed from those who reported an unmet need for help in marital status (married: 76.5% vs. 62.2%), food insecurity (18.6% vs. 36.1%), missed appointments due to transportation (7.9% vs. 28.3%), dementia (7.2% vs. 13.1%), depression (24.4% vs. 38.1%), and being homebound (14.5% vs. 23.7%).

### 3.3. Prevalence of Combinations of LTC Problems

**Figure 2:**
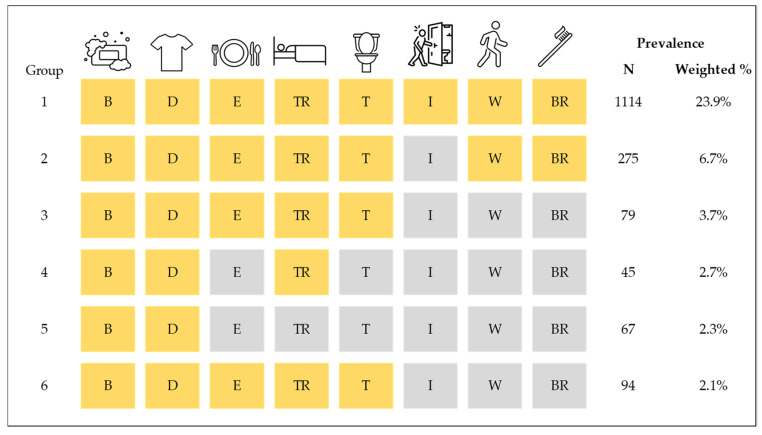
Prevalent combinations of activities of daily living (ADLs) among Veterans 65 years old or older. Data is from the 2021 HERO CARE survey (N = 3985 Veterans with at least one reported ADL problem). Yellow shading represents a reported problem or difficulty performing that ADL. The ADLs are as follows: B: bathing, D: dressing, E: eating, TR: transferring in/out of bed, T: toileting, I: managing incontinence, W: walking across a small room, and BR: brushing teeth.

We found 485 unique combinations of ADL problems with frequencies per combination group ranging from 1 to 1114 Veterans. Figure 2 presents the observed prevalence of the most frequently occurring combinations of ADLs. In group 1, almost a quarter of Veterans with ADL problems reported having a problem with all eight ADLs (23.9%), which is considered complete limitation with ADLs. The second most prevalent combination group (group 2) included seven ADLs and excluded managing incontinence (6.7%), followed by group 3 including five ADLs—bathing, dressing, eating, transferring, and using the toilet (3.7%). Bathing, dressing, eating, transferring, and toileting were included in four out of six of the most prevalent combinations.

**Figure 3:**
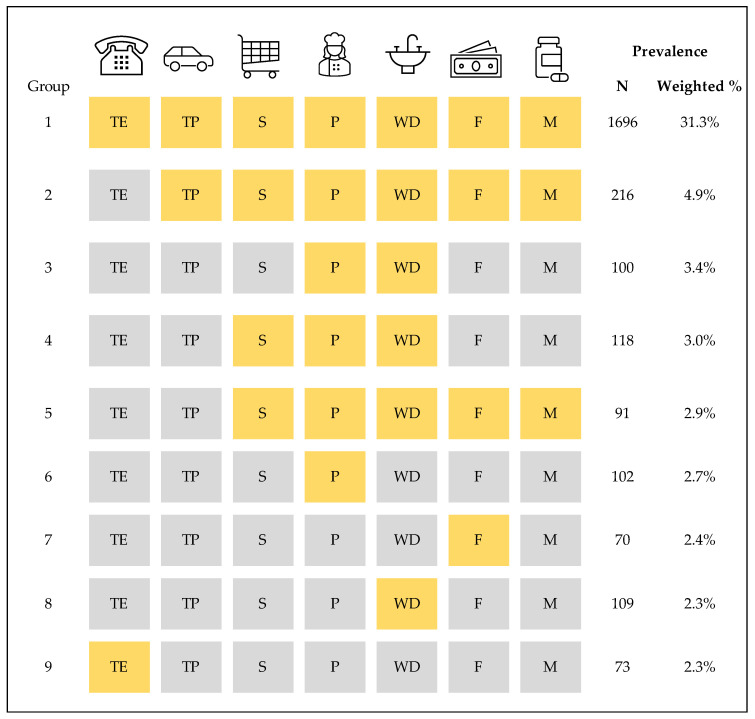
Prevalent combinations of instrumental activities of daily living (IADLs) among Veterans 65 years old or older. Data is from the 2021 HERO CARE survey (N = 4720 Veterans with at least one reported IADL problem). Yellow shading represents a reported problem or difficulty performing that IADL. The IADLs are as follows: TE = using the telephone; TP = transportation; S = grocery or other forms of shopping; P = preparing meals; WD = washing dishes; F = finances, including handling money or paying bills; and M = taking medicine at the right time/dose.

We found 404 unique combinations of IADL problems with frequencies per combination group ranging from 1 to 1696 Veterans. Figure 3 presents the observed prevalence of the most frequently occurring combinations of IADLs. In group 1, over a quarter of Veterans with IADL problems reported having a problem with all seven IADLs (31.3%), which indicated complete limitation with IADLs. The second most prevalent combination group (group 2) included six IADLs and excluded using the telephone (4.9%), followed by group 3 including two IADLs—preparing meals and washing dishes (3.4%). Problems with washing dishes and preparing meals were included in six out of nine of the most prevalent combinations.

## 4. Discussion

This study is the first to provide findings on how sociodemographic characteristics and health factors differ among Veterans by levels of the hierarchy of ADL and IADL problems and by the degree of need for help and to describe the combination patterns of ADLs and IADLs. We found that over a third of Veterans with LTC problems reported problems performing both ADLs and IADLs, and about a third reported unmet needs. Our analysis reveals important variation in the type and degree of need across domains and subgroups. Notably, Veterans who reported multiple ADL problems still reported unmet needs despite them likely meeting thresholds for home and community-based service eligibility. This suggests that functional impairment alone does not fully account for whether Veterans receive adequate assistance, pointing to potential gaps in service delivery, caregiving capacity, or systemic accessibility. Furthermore, Veterans with both ADL and IADL unmet needs were more likely to also experience social and mental health unmet needs. Furthermore, reporting problems with all ADLs or IADLs is common among Veterans [55].

These findings have important implications. First, they suggest that aligning VA home and community-based services more closely with patterns of Veteran-reported needs could improve care targeting. For example, Veterans with multiple IADL limitations but few ADL limitations may still experience high unmet needs, which highlights the importance of non-medical support services such as meal preparation, transportation, and medication management. This challenges traditional models that prioritize physical dependency and may undervalue cognitive or logistical impairments. In turn, these results may help prioritize outreach strategies by segmenting Veterans into high-need groups based on domain-specific difficulty patterns.

Although most older adults will need LTC at some stage in their life, it is important to better understand how much and what type of care is needed. In terms of the types of problems reported, our results show that ADLs and IADLs exist as clusters, as expected based on prior studies exploring patterns of ADL and IADL deficits [56]. Over a third of the Veterans reported co-occurring difficulties with dressing, bathing, eating, transferring, and toileting, the five most common ADLs that both the VA and Medicare use for assessing LTSS eligibility. Typically, the VA requires dependence with two or more ADLs to qualify for specific benefits like a homemaker/home health aide.

Understanding the resources available for assistance with IADLs may be challenging because VA guidelines generally do not define IADL assistance as medical expenses; however, it does recognize them as medical expenses for monthly VA pension or compensation. Furthermore, VA and Medicare differ in their definition of what constitutes as “medical care” and whether ADLs and IADLs are included. These varying definitions that impact the eligibility for benefits pose a barrier to older adults understanding and receiving assistance. Identifying the patterns of ADLs and IADLs among Veterans provides valuable information to align VA programs and services with Veterans’ needs. The insights and best practices that may arise from understanding the needs of Veterans can lead to the development of best practices that are applicable to the wider aging population, thereby improving care and support for all older adults.

Compared to studies in non-Veteran older adult populations, the proportion of unmet need in our sample appears similar to that of national estimates. For instance, a prior study found that approximately 30% of older Medicare beneficiaries with ADL or IADL difficulties reported at least one unmet need [57]. Nonetheless, the persistence of unmet needs highlights opportunities to further strengthen the adequacy, reach, and coordination of VA programs.

A report by the Government Accountability Office noted the need for the VA to develop measurable goals to address the challenges with Veteran access to long-term care, part of which includes aligning VA programs and services with Veterans’ needs [4,58]. Our findings are intended to provide estimates of the prevalence of LTC needs and unmet needs to effectively inform plans to meet the escalating demand for long-term care. Based on our estimates, about one-sixth of Veterans aged 65 and over and a third of those with LTC needs reported unmet needs for help which could potentially be addressed by home and community-based services.

### 4.1. Strengths

Our study has many strengths and implications. The HERO CARE survey is the first VHA multicenter survey aimed specifically at assessing the needs and unmet needs of Veterans across multiple domains. Our weighted estimates are representative of the Veteran population at the five sites and generalizable to the Veteran population of these five sites. To evaluate the generalizability of our survey findings, we compared key demographic characteristics including age, sex, marital status, rurality, and race and ethnicity between our target sample (all community-dwelling Veterans at the sites surveyed), the weighted sample, and the national population of VHA-enrolled Veterans. We observed that our weighted sample resembled the target sample on age, sex, race and ethnicity, and rurality. Our weighted sample and the target sample resembled the national population of VHA-enrolled Veterans on age and sex. However, our target sample and the weighted sample had a higher proportion of Hispanic Veterans compared to national estimates (17.1% vs. 18.9% vs. 9.0%, respectively). The large sample size across five sites surveyed and weighted estimates is a key strength for the generalizability of our study. Also, our ability to combine survey data with VHA EHR data and CMS data offers us the unique ability to include healthcare utilization data and diagnosed conditions that are not self-reported, reducing misclassification bias.

Furthermore, unmet need is not routinely measured, and although the utilization of services and supports may be used as a proxy for meeting needs, it does not provide information on how well needs are met. Veterans may use VHA services and still have unmet needs if there is a mismatch between their level of need and the services they are using or the availability, accessibility, affordability, acceptability, and adequacy of services and resources provided [59]. Hence, a strength of our study is that Veterans’ perception of unmet needs for assistance is available in the data.

Additionally, our findings point to the need for further research on ADL and IADL unmet needs to help policymakers make informed evidence-based decisions concerning home and community-based services and LTSS. Our research is timely and meets the priorities set by the VA Office of Research and Development HSR&D program and the VHA including increasing the awareness of the role social determinants play in health and health equity and specifically related to long-term care, aging, and support services. There is a need for research on how aging affects the care required and the ability to self-care [60].

A goal of the Department of Veterans Affairs’ Equity Action Plan is to address health equity for underserved Veterans by addressing the social and economic determinants of equity for underserved Veterans across socioeconomic factors to include race, ethnicity, gender, income, education, and life experience, which impact access and quality care [61]. On a similar vein, the priorities for the 2022–2032 CMS Framework for Health Equity ask for the expansion of data collecting, reporting, and analysis to identify the causes of disparities and address inequities [62]. One of the priorities involves advancing language access, health literacy, and the provision of culturally tailored services. On target with CMS priorities, our findings show that Veterans with unmet needs were more likely to report low health literacy and belong to a racial or ethnic minority group, necessitating the provision of culturally tailored services.

These findings are valuable in informing areas for reducing inequities, setting targets for reducing those inequities, resource and intervention development and allocation, and considering how to make investments in key populations with especially large disparities in needs.

### 4.2. Limitations

This study has some limitations. One is non-response bias at either the unit and/or item level. The overall response rate (40.3%) of the HERO CARE survey is comparable to other mailed national surveys; however, differences may exist between respondents and non-respondents. Self-reported data may be subject to recall bias, as many of our survey questions were phrased as “In the last month” or in “the last 12 months,” and social desirability bias, potentially leading to the under- or over-reporting of problems and unmet needs.

Furthermore, the measure of unmet need is complex. Needs may be fully met, partially met, met with delay or difficulty, or met inconsistently. Further complicating the measure of unmet need is the varying definitions of unmet need depending on the types of needs [63,64]. In this study, as well as most studies of unmet needs in general, self-reports of unmet needs do not provide information on the reason for unmet needs, such as supply and demand and access issues. The lack of standardized data and definitions across studies to measure different types of unmet needs limits the generalizability of our findings to other populations outside of the VHA.

## 5. Conclusions

Our findings show that Veterans’ social determinants of health and clinical characteristics differ by a hierarchy of problems with ADLs and IADLs and by the degree of need for help. They also reveal that Veterans have a high prevalence of limitation with all ADLs or IADLs. The implications of our findings are direct and will help inform programs, interventions, and policies related to unmet needs among both Veterans and non-Veterans. These data are aligned with both VA and non-VA research priorities, and the results may help advance health equity by identifying health disparities in unmet needs. Future work will focus on validating self-reported needs and unmet needs using VHA electronic health records and examining patterns of unmet needs among Veterans actively receiving specific VA services to better assess alignment between needs and service delivery.

## Figures and Tables

**Table 1:** Characteristics of Veterans by hierarchy of ADL and IADL problems.

							Post hoc Pairwise Comparisons					
	Overall	0	1	2	3	*p*	0,1	0,2	0,3	1,2	1,3	2,3
		No ADL/ IADL Problems	IADL Problems Only	ADL Problems Only	Both ADL and IADL Problems		No ADL/ IADL vs. IADL Only	No ADL/ IADL vs. ADL Only	No ADL/ IADL vs. Both	IADL Only vs. ADL Only	IADL Only vs. Both	ADL Only vs. Both
N, Weighted %	N = 7424, Weighted N = 362,639	N = 2379, 47.5%	N = 1060, 13.2%	N = 325, 5.6%	N = 3660, 33.7%							
**Sociodemographic Characteristics**												
**Age Mean (SE)**	76.5 (0.22)	75.7 (0.32)	77.4 (0.55)	75.4 (0.75)	77.6 (0.38)	**<0.001**	0.01	0.76	**<0.001**	0.04	0.71	0.01
**Sex**						0.41	---	---	---	---	---	---
Male	7240 (95.8%)	2338 (96.9%)	1029 (93.7%)	314 (93.6%)	3559 (95.4%)							
Female	181 (4.2%)	41 (3.1%)	31 (6.3%)	11 (6.4%)	98 (4.6%)							
**Race and** **Ethnicity**						**<0.001**	0.11	0.48	**<0.001**	0.16	0.67	0.04
Non-Hispanic White	5704 (69.5%)	1918 (75.0%)	842 (64.8%)	266 (78.6%)	2678 (61.9%)							
Non-Hispanic Black	520 (10.5%)	150 (10.2%)	59 (13.1%)	20 (5.1%)	291 (10.9%)							
Hispanic	628 (14.4%)	138 (9.2%)	82 (16.6%)	20 (11.0%)	388 (21.4%)							
Other	387 (5.6%)	117 (5.5%)	54 (5.5%)	11 (4.4%)	205 (5.8%)							
**Marital Status**						0.05	---	---	---	---	---	---
Married	4167 (64.4%)	1245 (62.2%)	621 (68.8%)	185 (76.0%)	2116 (64.0%)							
Single Never Married	203 (4.0%)	68 (3.6%)	30 (6.3%)	9 (3.8%)	96 (3.7%)							
Separated, Divorced, or Widowed	3048 (31.6%)	1064 (34.2%)	407 (24.9%)	131 (20.2%)	1446 (32.8%)							
**Education Level**						**0.01**	0.12	0.05	**0.004**	0.3	0.84	0.12
Less than or equal to HS	2361 (30.7%)	658 (25.8%)	360 (34.5%)	99 (37.9%)	1244 (35.2%)							
Some college/Associates	2431 (37.7%)	837 (39.8%)	328 (35.8%)	99 (25.1%)	1167 (37.5%)							
Bachelor’s/Graduate School	2013 (31.6%)	696 (34.4%)	281 (29.8%)	97 (37.1%)	939 (27.3%)							
**Rurality**						0.69	---	---	---	---	---	---
Urban	5957 (80.3%)	1901 (81.4%)	862 (80.8%)	248 (76.4%)	2946 (79.3%)							
Rural	1465 (19.7%)	477 (18.6%)	197 (19.2%)	77 (23.6%)	714 (20.7%)							
**Area Deprivation Index**						**0.01**	0.73	0.42	**<0.001**	0.67	0.1	0.68
Quantile 1	1084 (9.7%)	370 (10.4%)	156 (8.0%)	51 (11.9%)	507 (9.0%)							
Quantile 2	1786 (22.6%)	595 (25.4%)	282 (24.6%)	77 (23.6%)	832 (17.7%)							
Quantile 3	1774 (24.0%)	589 (26.2%)	252 (24.3%)	70 (17.6%)	863 (21.9%)							
Quantile 4	1415 (20.7%)	422 (19.9%)	194 (21.6%)	55 (20.3%)	744 (21.5%)							
Quantile 5	1309 (23.0%)	381 (18.2%)	170 (21.6%)	68 (26.7%)	690 (29.9%)							
**Has Medication Insecurity**						**0.04**	0.04	0.6	**0.003**	0.63	0.68	0.49
No	6988 (94.1%)	2287 (96.7%)	988 (93.3%)	311 (94.8%)	3402 (91.0%)							
Yes	323 (5.9%)	57 (3.3%)	57 (7.7%)	8 (5.2%)	201 (9.0%)							
**Has Food** **Insecurity**						**<0.001**	**<0.001**	0.4921	**<0.001**	**<0.001**	**0.004**	**<0.001**
No	6261 (83.6%)	2158 (92.6%)	905 (81.2%)	288 (94.0%)	2910 (70.2%)							
Yes	1065 (16.4%)	186 (7.4%)	144 (18.8%)	31 (6.0%)	704 (29.8%)							
**Has Low Health Literacy**						**<0.001**	**<0.001**	0.32	**<0.001**	**0.002**	0.02	**<0.001**
No	3930 (67.1%)	1862 (84.2%)	539 (56.3%)	249 (79.2%)	1280 (45.3%)							
Yes	3285 (32.9%)	443 (15.8%)	495 (43.7%)	68 (20.8%)	2279 (54.7%)							
**Has Missed** **Appointments**						**<0.001**	**<0.001**	0.69	**<0.001**	**<0.001**	0.1	**<0.001**
No	6498 (91.6%)	2292 (97.9%)	944 (88.4%)	310 (97.5%)	2952 (82.8%)							
Yes	891 (8.4%)	82 (2.1%)	111 (11.6%)	15 (2.5%)	683 (17.2%)							
**Clinical Characteristics**												
**Has Substance Use Disorder**						**0.001**	0.04	**0.002**	**<0.001**	0.07	0.47	0.15
No	7109 (96.6%)	2298 (97.9%)	1010 (96.4%)	307 (91.4%)	3494 (95.7%)							
Yes	315 (3.4%)	81 (2.1%)	50 (3.6%)	18 (8.6%)	166 (4.3%)							
**Has Dementia**						**<0.001**	**<0.001**	0.077	**<0.001**	**<0.001**	**<0.001**	**<0.001**
No	6172 (96.0%)	2247 (99.3%)	890 (96.2%)	301 (98.8%)	2734 (91.0%)							
Yes	1173 (4.0%)	99 (0.7%)	154 (3.8%)	23 (1.2%)	897 (9.0%)							
**Depression (PHQ-2)**						**<0.001**	**<0.001**	0.2161	**<0.001**	0.16	0.03	**0.003**
0–2	5741 (82.4%)	2201 (92.2%)	882 (78.9%)	275 (87.4%)	2383 (68.9%)							
≥3	1530 (17.6%)	146 (7.8%)	161 (21.1%)	47 (12.6%)	1176 (31.1%)							
**Anxiety (GAD-2)**						**<0.001**	**0.002**	0.57	**<0.001**	0.25	0.02	0.01
0–2	6175 (86.0%)	2214 (93.6%)	920 (84.7%)	289 (91.4%)	2752 (74.9%)							
≥3	1056 (14.0%)	119 (6.4%)	117 (15.3%)	28 (8.6%)	792 (25.1%)							
**VA-CMS measures**												
**CMS-HCC risk score Mean (SE)**	1.26 (0.02)	0.99 (0.03)	1.18 (0.05)	1.14 (0.08)	1.67 (0.05)	**<0.001**	**0.001**	0.09	**<0.001**	0.64	**<0.001**	**<0.001**
**Service-connected Mean (SE)** **^a^**	55.2 (1.2)	44.8 (2.0)	62.7 (2.6)	60.6 (3.6)	63.4 (1.7)	**<0.001**	**<0.001**	**<0.001**	**<0.001**	0.64	0.83	0.49
**Service-connected rating**						**<0.001**	**<0.001**	0.02	**<0.001**	0.99	0.19	0.45
Not service-connected	3675 (44.6%)	1316 (50.3%)	543 (41.7%)	152 (40.3%)	1664 (38.3%)							
≤40%	1312 (20.0%)	493 (24.4%)	185 (15.6%)	61 (16.8%)	573 (16.2%)							
50–90%	1670 (26.2%)	425 (20.3%)	248 (33.8%)	85 (34.7%)	912 (30.1%)							
100%	767 (9.2%)	145 (5.0%)	84 (8.9%)	27 (8.2%)	511 (15.4%)							
** Dependency Characteristics **												
**Has a Caregiver**						**<0.001**	**<0.001**	**<0.001**	**<0.001**	0.46	**<0.001**	**<0.001**
No	3392 (65.9%)	1871 (88.9%)	489 (62.9%)	199 (68.5%)	833 (34.7%)							
Yes	3805 (34.1%)	415 (11.1%)	537 (37.1%)	116 (31.5%)	2737 (65.3%)							
**Homebound** **Status**						**<0.001**	0.01	0.033	**<0.001**	0.02	**<0.001**	**<0.001**
Not Homebound	6288 (91.4%)	2315 (98.0%)	977 (93.4%)	301 (97.8%)	2695 (80.1%)							
Homebound	1061 (8.6%)	51 (2.0%)	75 (6.6%)	20 (2.2%)	915 (19.9%)							
**Total ADL problems Mean (SE)**	1.88 (0.08)	NA	NA	1.81 (0.14)	5.26 (0.10)	**<0.001**	---	---	---	---	---	**<0.001**
**Total IADL Problems Mean (SE)**	2.04 (0.07)	NA	2.43 (0.1)	NA	5.11 (0.09)	**<0.001**	---	---	---	---	<0.001	---

Table 1 Notes: ^a^ Service-connected mean calculated among Veterans with a service-connected condition. ADLs: activities of daily living; IADLs: instrumental activities of daily living; PHQ2: 2-item patient health questionnaire (score range, 0–6); GAD2: 2-item generalized anxiety disorder questionnaire (score range, 0–6); PHQ2 and GAD2 scores ≥ 3 indicate possible depression or anxiety; CMS: Centers for Medicare and Medicaid Services; HCC: hierarchical condition category. Bold values indicate significance at <0.05 for an omnibus *p*-value or <0.008 for post hoc pairwise comparisons with the Bonferroni correction.

**Table 2:** Characteristics of Veterans by degree of need for help with ADLs and IADLs.

						*p*	Post hoc Pairwise Comparisons					
	Overall	0	1	2	3		0,1	0,2	0,3	1,2	1,3	2,3
		No ADL/ IADL Problems	Has ADL or IADL Problems (N = 5045, 52.5% ^a^)				No ADL/ IADL vs. Self- Manage	No ADL/ IADL vs. Sufficient Help	No ADL/ IADL vs. Unmet Need	Self-Manage vs. Sufficient Help	Self- Manage vs. Unmet Need	Sufficient Help vs. Unmet Need
			Self- Manage	Sufficient Help	Unmet Need							
Unweighted N	N = 7424	N = 2379	N = 1160	N = 1770	N = 2115							
Weighted % among whole sample ^a^		47.5% ^a^	19.0% ^a^	16.6% ^a^	16.9% ^a^							
Weighted % among with problem ^b^		-----	36.1% ^b^	31.6% ^b^	32.3% ^b^							
Sociodemographic Characteristics												
**Age Mean (SE)**	76.5 (0.22)	75.7 (0.32)	75.0 (0.48)	78.9 (0.38)	78.4 (0.52)	**<0.001**	0.25	**<0.001**	**<0.001**	**<0.001**	**<0.001**	0.43
**Sex**												
Male	7240 (95.8%)	2338 (96.9%)	1112 (89.5%)	1734 (98.3%)	2056 (97.2%)	**<0.001**	**0.001**	0.16	0.06	**<0.001**	**<0.001**	0.23
Female	181 (4.2%)	41 (3.1%)	48 (10.5%)	36 (1.7%)	56 (2.8%)							
**Race and** **Ethnicity**						**<0.001**	0.06	**<0.001**	**<0.001**	0.71	0.26	0.02
NHW	5704 (69.5%)	1918 (75.0%)	921 (67.4%)	1373 (66.9%)	1492 (58.6%)							
NHB	520 (10.5%)	150 (10.2%)	76 (9.7%)	104 (7.0%)	190 (15.7%)							
Hispanic	628 (14.4%)	138 (9.2%)	85 (17.6%)	149 (20.8%)	256 (19.4%)							
Other	387 (5.6%)	117 (5.5%)	55 (5.3%)	96 (5.2%)	119 (6.2%)							
**Marital** **Status**						**<0.001**	0.81	**<0.001**	0.19	**<0.001**	0.61	**<0.001**
Married	4167 (64.4%)	1245 (62.2%)	537 (61.5%)	1138 (76.5%)	1247 (62.2%)							
Single Never Married	203 (4.0%)	68 (3.6%)	38 (4.7%)	43 (1.7%)	54 (6.7%)							
Separated, Divorced or Widowed	3048 (31.6%)	1064 (34.2%)	583 (33.9%)	588 (21.8%)	813 (31.0%)							
**Education Level**						**0.01**	0.32	**0.002**	0.01	0.33	0.48	0.68
Less than or equal to HS	2361 (30.7%)	658 (25.8%)	348 (31.7%)	646 (38.6%)	709 (35.9%)							
Some college/Associates	2431 (37.7%)	837 (39.8%)	389 (36.5%)	559 (33.5%)	646 (37.1%)							
Bachelor’s/Graduate School	2013 (31.6%)	696 (34.4%)	301 (31.8%)	440 (27.8%)	576 (27.0%)							
**Rurality**						0.3	---	---	---	---	---	---
Urban	5957 (80.3%)	1901 (81.4%)	902 (79.0%)	1409 (76.5%)	1745 (82.5%)							
Rural	1465 (19.7%)	477 (18.6%)	258 (21.0%)	361 (23.5%)	369 (17.5%)							
**Area Deprivation Index**						**<0.001**	0.09	**<0.001**	**0.002**	0.06	0.14	0.43
Quantile 1	1084 (9.7%)	370 (10.4%)	166 (8.6%)	225 (8.6%)	323 (10.1%)							
Quantile 2	1786 (22.5%)	595 (25.4%)	262 (23.6%)	446 (19.4%)	483 (16.7%)							
Quantile 3	1774 (24.0%)	589 (26.2%)	276 (19.1%)	436 (23.4%)	473 (24.1%)							
Quantile 4	1415 (20.7%)	422 (19.9%)	229 (24.9%)	356 (17.2%)	408 (21.6%)							
Quantile 5	1309 (23.0%)	381 (18.2%)	218 (23.9%)	297 (31.4%)	413 (27.6%)							
**Has** **Medication** **Insecurity**						**0.008**	0.03	0.13	**<0.001**	0.47	0.54	0.12
No	6988 (94.1%)	2287 (96.7%)	1076 (91.7%)	1678 (93.8%)	1947 (89.7%)							
Yes	323 (5.9%)	57 (3.3%)	68 (8.3%)	66 (6.2%)	132 (10.3%)							
**Has Food** **Insecurity**						**<0.001**	**<0.001**	**<0.001**	**<0.001**	0.82	**<0.001**	**<0.001**
No	6261 (83.6%)	2158 (92.6%)	955 (80.6%)	1537 (81.4%)	1611 (63.9%)							
Yes	1065 (16.4%)	186 (7.4%)	192 (19.4%)	212 (18.6%)	475 (36.1%)							
**Has Low Health** **Literacy**						**<0.001**	**<0.001**	**<0.001**	**<0.001**	**<0.001**	**<0.001**	0.06
No	3930 (67.1%)	1862 (84.2%)	793 (71.0%)	707 (44.7%)	568 (36.6%)							
Yes	3285 (32.9%)	443 (15.8%)	341 (29.0%)	1013 (55.3%)	1488 (63.4%)							
**Has Missed Appointments**						**<0.001**	**<0.001**	**<0.001**	**<0.001**	0.7	**<0.001**	**<0.001**
No	6498 (91.6%)	2292 (97.9%)	1080 (93.0%)	1575 (92.1%)	1551 (71.7%)							
Yes	891 (8.4%)	82 (2.1%)	74 (7.0%)	185 (7.9%)	550 (28.3%)							
**Clinical Characteristics**												
**Has Substance Use Disorder**						**<0.001**	0.05	0.05	**<0.001**	0.63	0.22	0.02
No	7109 (96.6%)	2298 (97.9%)	1105 (95.9%)	1702 (96.6%)	2004 (93.7%)							
Yes	315 (3.4%)	81 (2.1%)	55 (4.1%)	68 (3.4%)	111 (6.3%)							
**Dementia**						**<0.001**	0.16	**<0.001**	**<0.001**	**<0.001**	**<0.001**	**<0.001**
No	6172 (96.0%)	2247 (99.3%)	1094 (99.0%)	1390 (92.8%)	1441 (86.9%)							
Yes	1173 (4.0%)	99 (0.7%)	55 (1.0%)	363 (7.2%)	656 (13.1%)							
**Depression (PHQ-2)**						**<0.001**	**<0.001**	**<0.001**	**<0.001**	0.14	**<0.001**	**<0.001**
0–2	5741 (82.4%)	2201 (92.2%)	954 (81.7%)	1364 (75.6%)	1222 (61.9%)							
≥3	1530 (17.6%)	146 (7.8%)	185 (18.3%)	369 (24.4%)	830 (38.1%)							
**Anxiety (GAD-2)**						**<0.001**	**0.002**	**<0.001**	**<0.001**	0.33	**0.001**	0.01
0–2	6175 (86.0%)	2214 (93.6%)	1002 (84.9%)	1473 (80.9%)	1486 (70.8%)							
≥3	1056 (14.0%)	119 (6.4%)	127 (15.1%)	250 (19.1%)	560 (29.2%)							
**VA and CMS measures**												
**HCC risk score Mean (SE)**	1.26 (0.02)	0.99 (0.03)	1.13 (0.04)	1.60 (0.06)	1.78 (0.08)	**<0.001**	**0.008**	**<0.001**	**<0.001**	**<0.001**	**<0.001**	0.06
**Service-connected Mean (SE)** **^c^**	55.2 (1.2)	44.8 (2.0)	59.5 (2.2)	66.2 (2.4)	63.7 (2.2)	**<0.001**	**<0.001**	**<0.001**	**<0.001**	0.04	0.17	0.44
**Service-connected Rating**						**<0.001**	**<0.001**	**<0.001**	**<0.001**	0.01	**0.006**	0.96
Not service connected	3675 (44.6%)	1316 (50.3%)	561 (37.5%)	851 (39.4%)	947 (41.5%)							
≤ 40%	1312 (20.0%)	493 (24.4%)	217 (18.2%)	277 (15.0%)	325 (14.8%)							
50–90%	1670 (26.2%)	425 (20.3%)	292 (37.0%)	430 (29.2%)	523 (27.6%)							
100%	767 (9.2%)	145 (5.0%)	90 (7.3%)	212 (16.4%)	320 (16.0%)							
**Dependency Characteristics**												
**Has a** **Caregiver**						**<0.001**	**<0.001**	**<0.001**	**<0.001**	**<0.001**	**<0.001**	0.37
No	3392 (65.9%)	1871 (88.9%)	771 (73.1%)	409 (31.7%)	341 (27.9%)							
Yes	3805 (34.1%)	415 (11.1%)	350 (26.9%)	1316 (68.3%)	1724 (72.1%)							
**Homebound Status**						**<0.001**	0.01	**<0.001**	**<0.001**	0.04	**<0.001**	**0.003**
Not Homebound	6288 (91.4%)	2315 (98.0%)	1099 (93.1%)	1439 (85.5%)	1435 (76.3%)							
Homebound	1061 (8.6%)	51 (2.0%)	51 (6.9%)	316 (14.5%)	643 (23.7%)							
**Hierarchy of ADL and IADL problems**												
No problems	2379 (47.5%)	2379 (100%)	0 (0%)	0 (0%)	0 (0%)	---	---	---	---	---	---	---
Only IADL Problems	1060 (13.2%)	0 (0%)	314 (26.6%)	510 (31.5%)	236 (17.2%)							
Only ADL Problems	325 (5.6%)	0 (0%)	264 (25.6%)	32 (3.1%)	29 (1.2%)							
Both ADL and IADL Problems	3660 (33.7%)	0 (0%)	582 (47.9%)	1228 (65.4%)	1850 (81.6%)							
**Total ADL Problems Mean (SE)**	1.88 (0.08)	---	2.71 (0.21)	3.37 (0.19)	4.47 (0.17)	**<0.001**	---	---	---	0.02	**<0.001**	**<0.001**
**Total IADL problems Mean (SE)**	2.04 (0.07)	---	2.61 (0.18)	4.14 (0.14)	5.10 (0.12)	**<0.001**	---	---	---	**<0.001**	**<0.001**	**<0.001**
**Total ADL Unmet Needs Mean (SE)**	---	---	---	---	1.32 (0.09)	---	---	---	---	---	---	---
**Total IADL Unmet Needs Mean (SE)**	---	---	---	---	2.56 (0.09)	---	---	---	---	---	---	---

Table 2 Notes: ADLs: activities of daily living; IADLs: instrumental activities of daily living; PHQ2: 2-item patient health questionnaire (score range, 0–6); GAD2: 2-item generalized anxiety disorder questionnaire (score range, 0–6); PHQ2 and GAD2 scores ≥ 3 indicate possible depression or anxiety. Bold values indicate significance at <0.05 for an omnibus *p*-value or <0.008 for post hoc pairwise comparisons with the Bonferroni correction. ^a^ denominator is N = 7424. ^b^ denominator is N = 5045. ^c^ Service-connected mean calculated among Veterans with a service-connected condition.

## Data Availability

The data sets generated and analyzed during this study are not publicly available owing to the Department of Veterans Affairs and ethical redactions as well as the redaction of ethically sensitive information; however, instructions on obtaining the data are available upon request.

## Abbreviations

The following abbreviations are used in this manuscript:

VHA	Veterans Health Administration
VA	United States Department of Veterans Affairs
LTSS	Long-term services and supports
LTC	Long-term care
ADLs	Activities of daily living
IADLs	Instrumental activities of daily living
PLI	Predicted long-term institutionalization score
CMS	Centers for Medicaid and Medicare Services
HCCs	Hierarchical condition categories
PHQ-2	Patient health questionnaire 2-item
GAD-2	Generalized anxiety disorder questionnaire 2-item
EHR	Electronic heath record
